# Papers by Medical Officers

**Published:** 1918-10

**Authors:** 


### THE PUBLICATION OF PAPERS BY MEDICAL OFFICERS OF THE A. E. F.

Medical officers of the American Expeditionary Forces have been working in the war zone long enough to have had considerable experience in dealing with the problems relating to military surgery.

Since many of the best trained men in the United States, representing all the branches of medicine, are with the American Army, and since they are giving the best that is in them to their work with sick and wounded soldiers, it is to be expected that they have made observations which have enabled them to improve upon medical methods and surgical technic, and that they have made advances in sanitation and preventive medicine which, when recorded and published, will be valuable contributions to the sum of medical knowledge.

The Spanish-American War, brief as it was, and though it was an insignificant affair compared to the present world conflict, set many of the ablest minds in American medicine to work to solve the problems of yellow fever, malaria, typhoid fever, and dysentery, diseases which had caused many more deaths among United States troops than Spanish bullets. As a result, the transmission of yellow fever by the stegomyia fasciata mosquito was proved; practical methods for preventing malaria were evolved; and the house fly was convicted of carrying typhoid fever and dysentery. These and many other valuable contributions were made to medicine in the Spanish-American War, which in their far reaching effects have more than compensated for the expense and the man loss in that conflict.

The French and British contributions to the science and literature of war medicine and surgery during the past four years have been epoch making in many respects; and a number of American surgeons and research workers have already reported original investigations and improvements of accepted methods that have been recognized as great advances in the treatment of wounds and in the prevention of disease. War medical literature, however, by American medical officers has not been very prolific up to this time.

### Duty of Medical Officers to Contribute to the Medical Literature of the War.

It cannot be the lot of every army surgeon to make great discoveries, but each man who is dealing with the medical and surgical problems of the war should not only do his very best with present methods, but he should try to improve upon them. If he makes an observation that enables him to get better results in treating his patients, or if he makes an original investigation of value, it is his duty to make reports on his work and allow it to be published. Others may then have the opportunity of verifying his work and, if it proves to be of value, of applying the knowledge for the general welfare of the army.

Of course a medical officer should not rush into print unless he has something of value to publish; but if a physician has had a large or unusual experience which may be helpful to others, it is his duty to write it up and publish it in medical journals that will be read by his confreres. It is not always necessary to have something new and original before contributing an article to a medical journal; but if the experience of a physician or surgeon proves that an old method is better than new ones that have been advised, it is worth while publishing his observations.

If physicians waited until they had something entirely new and original to publish there would be few medical books and journals published. A well known medical editor reviewed a number of the leading medical journals of several countries and his conclusions were that less that five per cent of the matter in them was new or original. He did not think that this fact made medical journals less valuable, because they contain the sum of the experiences of the men who are accomplishing most in medicine; and often the reiteration, or the restating from a different view point of well known facts is production of practical results.

It is certain that medical officers with the American Expeditionary Forces will prepare many important papers for publication in medical journals; and a problem for consideration is how to get their work published at an early date and placed in the hands of their confreres who are dealing with the same problems. It is to be expected that most of the papers will be published in the medical journals of the United States, though a number have appeared in British journals. It is sometimes several months before a paper can be published in a medical journal, because such a publication usually has enough manuscript on hand for a number of issues, and the mail problem is a difficult one during the war.

### Abstracts and Reports in War Medicine.

*War Medicine* does not publish original articles, but it would like to be a medium for disseminating reports of the work of medical officers with the American Expeditionary Forces. It can and will publish abstracts of original articles and reports by medical officers before it would be possible to get them published in medical journals.

While all medical journals expect exclusive right of publishing the papers which appear in them, no editor objects to the publication of abstracts of papers either before or after they appear in his publication; and during the war when time is such an important element it is essential that the observations of army surgeons be published as soon as possible.

A circular from the Chief Surgeon's office, published on page 425, states that the Surgeon General at Washington has ruled that before a paper by a medical officer in the United States can be published it must first be sent to his office for approval. Therefore the proper procedure for a medical officer to follow, when he desires an article published, is to submit two copies to his commanding officer, who sends them with an endorsement to the Chief Surgeon, who in turn makes his endorsement and transmits them to the Surgeon General in Washington. If the Surgeon General approves the publication of the article, one copy is sent to the journal designated by the author and one copy is kept on file in the Surgeon General's Office.

According to a recent arrangement, the Chief Surgeon is allowing *War Medicine* to publish abstracts of original articles by medical officers so that there will be no delay in disseminating information contained in them among the physicians in the American Expeditionary Forces. Authors of such articles can be helpful to their confreres and at the same time add to the value of *War Medicine* if they will make an abstract of from 250 to 750 words of the articles and send two copies with the copies of the original articles to the Chief Surgeon. Often the author's abstract is of greater value than that of the most experienced medical reviewer.

Consultants, division surgeons, commanding officers of hospitals, chiefs of laboratories, and heads of other groups of medical men, who are engaged in practical or research work with the American Expeditionary Forces, would encourage medical officers to report their work, provided of course that they have something of value to publish. Medical officers should be made to feel that, if they have done worthy work and it is published, they are making history as much as the men who are on the firing line, and *War Medicine* would like the privilege of publishing at least the resume of the current medical and surgical history of the War as made by American physicians serving in France and England.

